# Identification of low-value practices susceptible to gender bias in primary care setting

**DOI:** 10.1186/s12875-024-02456-8

**Published:** 2024-06-08

**Authors:** Virtudes Pérez-Jover, Alicia Sánchez-García, Adriana Lopez-Pineda, Irene Carrillo, José Joaquín Mira, Concepción Carratalá-Munuera

**Affiliations:** 1 Department of Health Psychology, Miguel Hernandez University of Elche, Elche, Spain; 2 Department of Clinical Medicine, Miguel Hernandez University of Elche, San Juan de Alicante, Spain; 3 Network for Research on Chronicity, Primary Care, and Health Promotion (RICAPPS), San Juan de Alicante, Spain; 4 Atenea Research Group, Foundation for the Promotion of Health and Biomedical Research, San Juan de Alicante, Spain; 5 Alicante-Sant Joan d’Alacant Health Department, San Juan de Alicante, Spain

**Keywords:** Low-value practices, Gender bias, Adverse events, Primary care

## Abstract

**Background:**

Data on overuse of diagnostic and therapeutic resources underline their contribution to the decline in healthcare quality. The application of “Do Not Do” recommendations, in interaction with gender biases in primary care, remains to be fully understood. Therefore, this study aims to identify which low-value practices (LVPs) causing adverse events are susceptible to be applied in primary care setting with different frequency between men and women.

**Methods:**

A consensus study was conducted between November 1, 2021, and July 4, 2022, in the primary care setting of the Valencian Community, Spain. Thirty-three of the 61 (54.1%) health professionals from clinical and research settings invited, completed the questionnaire. Participants were recruited by snowball sampling through two scientific societies, meeting specific inclusion criteria: over 10 years of professional experience and a minimum of 7 years focused on health studies from a gender perspective. An initial round using a questionnaire comprising 40 LVPs to assess consensus on their frequency in primary care, potential to cause serious adverse events, and different frequency between men and women possibly due to gender bias. A second round-questionnaire was administered to confirm the final selection of LVPs.

**Results:**

This study identified nineteen LVPs potentially linked to serious adverse events with varying frequencies between men and women in primary care. Among the most gender-biased and harmful LVPs were the use of benzodiazepines for insomnia, delirium, and agitation in the elderly, and the use of hypnotics without a previous etiological diagnosis.

**Conclusions:**

Identifying specific practices with potential gender biases, mainly in mental health for the elderly, contributes to healthcare promotion and bridges the gap in gender inequalities.

**Trial registration:**

NCT05233852, registered on 10 February 2022.

**Supplementary Information:**

The online version contains supplementary material available at 10.1186/s12875-024-02456-8.

## Background

Over the past decades, there has been a growing focus in research on the issue of overdiagnosis and overtreatment in healthcare [1, 2]. This encompasses procedures, tests, and treatments that, due to their unnecessary or ineffective nature, not only pose significant physical harm to patients but also contribute to the wastage of money and resources. Identifying and addressing these low-value practices (LVP), characterized by greater risks and the availability of more cost-effective alternatives, is crucial for improving the overall quality and efficiency of healthcare [3]. Several efforts have been initiated to identify low-value care practices on a global scale: the ‘Choosing Wisely’ [4] campaign implemented in various countries, a compilation by the United Kingdom’s National Institute for Health and Care Excellence (NICE) [5], and other numerous lists containing 'Do not Do' recommendations have been compiled by scientific societies [6, 7] to guide healthcare professionals away from unnecessary or potentially harmful interventions.

Recent studies [8, 9] have documented frequent cases of overtreatment and overuse of medical interventions, where unnecessary procedures are performed, or medications are prescribed without clear benefit to the patient. According to Jungo et al. [10], up to 69% US older adults, with multiple chronic conditions and polypharmacy medication, use more than one inappropriate medication. Furthermore, García-Alegría et al. [11] highlights that rates of overtreatment vary significantly among different types of practice and geographic regions, emphasizing the complexity of this phenomenon and the need for specific strategies for mitigation. Despite these efforts, the issue persists. Therefore, it is essential to delve into understanding the underlying factors contributing to these practices to implement effective interventions and enhance the quality of healthcare.

Beyond the challenges of the de-implementation of LVPs, the influence of gender bias on clinical decision-making adds a layer of complexity to the healthcare landscape. Evidence suggests that biological and sociocultural differences between men and women can significantly impact healthcare delivery [12]. Previous studies [13, 14] reported a gender bias in clinical encounters, affecting areas such as the overuse of antibiotics for sore throat and pain management. This phenomenon could be due to factors like gender stereotypes influencing symptom perception and clinical decision-making, inadequate medical training on gender differences, a lack of evidence guiding treatment in women, or a higher rate of women’s attendance in primary care. These biases may lead to disparities in the application of medical interventions, potentially exposing one gender to a higher likelihood of overdiagnosis or overtreatment. While existing literature has begun to provide data of overuse, the application of “Do Not Do” recommendations, and gender biases in healthcare, there remains a notable gap in understanding how these factors intersect in the context of primary care. Understanding how gender biases intersect with the LVPs not only contributes to a more nuanced understanding of healthcare disparities but also lays the groundwork for targeted interventions to mitigate these biases.

Primary healthcare plays an important role in preventing, diagnosing, and treating illnesses. However, concerns about gender biases influencing the application of these recommendations are emerging in primary care setting [10, 15]. To explore clinical decision-making in primary care is needed, with a focus on gender biases and the challenges posed by the de-implementation of LVPs. Thus, the present study aimed to identify which LVPs causing adverse events are susceptible to be applied in primary care setting with different frequency between men and women.

## Methods

### Study design and participants

**Fig. 1:**
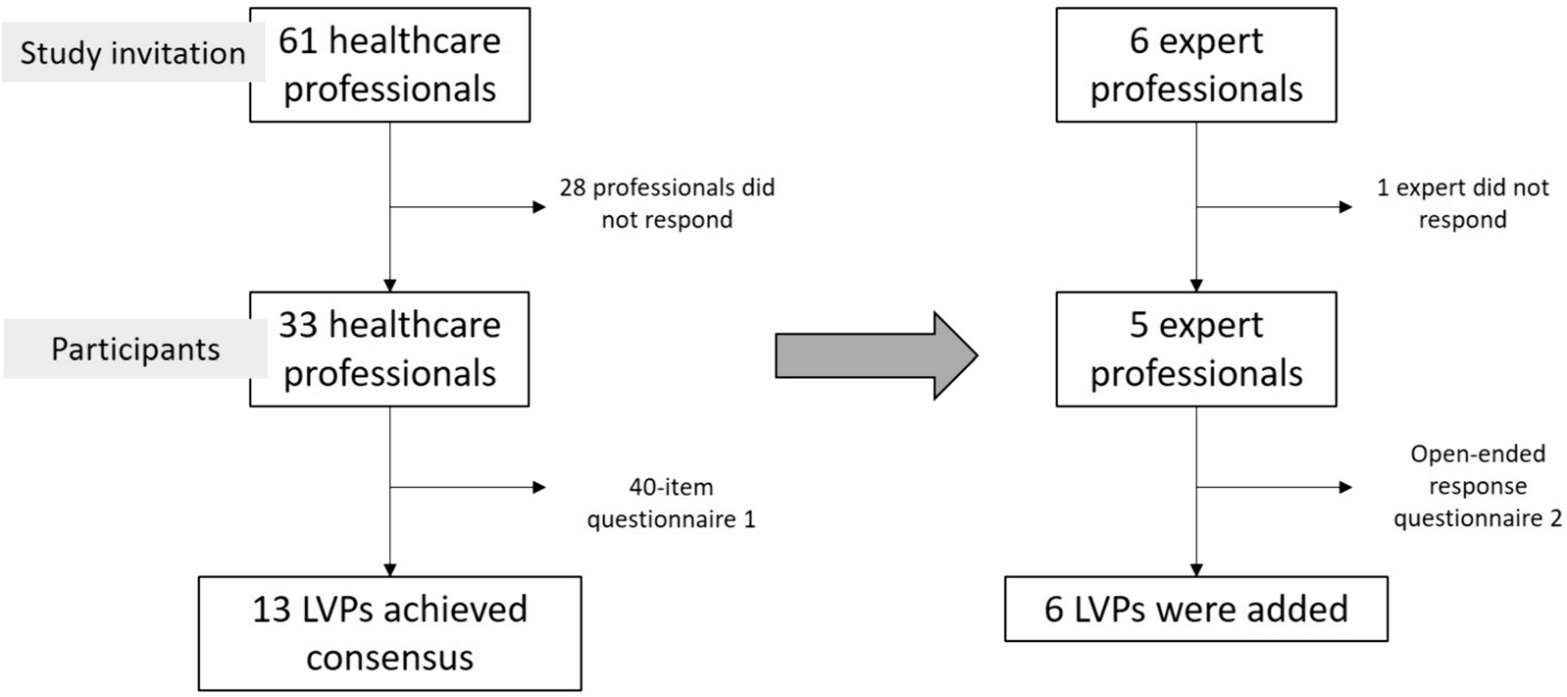
Study flowchart

This consensus study was carried out from November 1, 2021, to July 4, 2022 in primary care setting. This study is part of the OVERGEND Project, which was registered at ClinicalTrials.gov (NCT05233852) on February 10, 2022. The study protocol was approved by the corresponding institutional review board and published elsewhere [16]. The second round to achieve consensus was conducted using an open-ended questionnaire administered to the most expert participants given the high degree of consensus in acceptances and rejections during the first round. In this case, panelist outlined LVPs they believed had a significant negative impact on women’s health (Fig. 1).

A sample of 50 professionals was deemed sufficient to comprehensively explore both social and biological perspectives and achieve information saturation. In the initial phase, 61 health professionals from clinical and research settings were invited to participate in an online questionnaire, accounting for a potential 18% loss rate after agreeing to take part in the study. The inclusion criteria were having more than 10 years of professional experience (including both primary care and patient safety issues) and at least 7 years of experience in health studies from a gender perspective to be considered as expert in in this topic. Snowball sampling was conducted to recruit participants through two scientific societies and other contacts of the research group.

### Materials: questionnaires

The research group designed a first questionnaire with Likert scale multiple choice questions and a second questionnaire with open-ended questions. The questionnaire 1 included the LVPs that arise from those ‘Do Not Do’ recommendations aimed at preventing overuse as agreed upon by all the Spanish scientific societies [17] (included in the Commitment to Quality led by the Ministry of Health of Spain, and based on rigorous processes including exhaustive literature review, expert discussion, and evidence-based consensus, typically backed by current clinical experience and research), and met the following criteria:

- Recommendations of the societies implemented in primary care and other societies with recommendations with scope in this level of care.
- Ignoring the recommendation may lead to a serious avoidable adverse event (SAAE).

In order to help the researchers to identify these LVPs, a webinar with national experts was held on the topic ‘Overuse in primary care associated with gender bias’ [18]. In this webinar, experts debated the LVPs l most applied in primary care setting.

Finally, the questionnaire 1 consisted of 40 LVPs based on the selected ‘Do Not Do’ recommendations and, for each one, the participants assessed if it was susceptible to gender bias in primary care setting considering three aspects:

A. This LVP is still relatively frequent in primary care setting.
B. This practice could cause a SAAE to the patient.
C. The frequency of application of this practice is different between men and women probably for reasons of gender.

In the first step, participants were asked to rate their agreement or disagreement with each of these three statements in relation to each practice using a 11-point Likert scale, where 0 indicated total disagreement; 5, neither agreement nor disagreement; and 10, total agreement. Additional file 1 shows the questionnaire 1.

For the second step, a questionnaire was developed from the responses to the previous one. This included those LVPs, which reached the greatest level of general agreement among the professionals and experts were asked:

- Do you consider that these practices are susceptible to a gender bias in the primary care setting, increasing the risk of a SAAE in the population of a certain sex, and, therefore, are adequate to include in the review study of medical records (Overgend Project)? If not, which one would you exclude and why?

Following, the LVPs that reached the greatest level of agreement regarding the different frequency of application of the practice between men and women probably for reasons of gender, experts were asked:

- Do you consider that any of these practices are susceptible to a gender bias in the primary care setting, increasing the risk of a serious adverse event in the population of a certain sex? If yes, indicate which one and the reason.

### Procedure

An ad-hoc online platform was used (access by user and password) to collect the responses of the study participants of the first questionnaire. A letter of invitation was sent by email to the professionals selected by the research group and was provided a link to access the online questionnaire, a personal user and password. This letter included the study objectives, the potential use of the collected information, the instructions to participate in the study, the approximate process duration, the deadline to complete the questionnaire, and the acknowledgment of the research group. In addition, eligible professionals were informed that if they accessed the questionnaire and responded it, they consented to their participation in this study, and they could withdraw at any time. Their responses were anonymous; thus, no data can identify them if they were withdrawn. While the questionnaire was open, there was a call center (attention through e-mail and telephone) to attend to possible technical incidents.

The first questionnaire started on April 6, 2022 and was open until April 29, 2022. Participants received two reminders to increase the response rate.

After the analysis of the responses to the first questionnaire, the three respondents with the most experience in the fields of primary care and patient safety and the three respondents with the most experience in the field of gender research were selected by the researchers among the respondents and invited to respond to the second questionnaire. The second questionnaire was sent by email to these six participants on June 15, 2022 and they were given 2 weeks to complete the questionnaire.

### Data analysis

The quantitative data obtained from the first questionnaire were downloaded into an Excel sheet maintaining the participants’ confidentiality. For each item, we calculated the mean of the score, the coefficient of variation (CV), and the percentages of response categories (< 6, 6–7 and > 7 scores). The resulting score for each LVP was the weighted sum of the mean scores of each of the three items following the next equation: (A) + (1.3*B) + (1.6*C). The LVPs that exceed 20 points were considered that they reached the greatest level of general agreement. The LVPs that obtained a mean score of the item C higher than 3.0 were considered they reached the greatest level of agreement regarding the different frequency of application of the practice between men and women probably for reasons of gender. The analysis was carried out using the Microsoft Excel program (Microsoft, Redmond, WA, USA). For the analysis of the responses of the second questionnaire, the research group considered all the LVPs mentioned by the experts and the reasons, both to be excluded and to be included.

## Results

Of the 61 professionals who were invited to participate in the first questionnaire, 33 of them responded (54.1% response rate). The Supplementary Table 1 (Additional file 2) shows the results of the first questionnaire. Regarding the application of the LVPs in primary care setting, the LVPs number 14 and 30 achieved the highest level of agreement by professionals (mean score > 7, and at least 50% of participants agree). In regard to if LVPs could cause a SAAE, the LVPs number 8, 14, 25 and 34 achieved the highest level of agreement (mean score > 8, and at least 70% of participants agree). Regarding the different application of the LVPs between men and women due to reasons of gender, any LVP achieved a mean score higher than six, and the LVP number 14 achieved the highest score without achieving a consensus (30.3% and 33.3% of participants disagree and agree, respectively). Table 1 shows the LVPs that reached the greatest level of general agreement (30% (*n* = 12) of the total LVPs). Table 2 shows the LVPs that reached the greatest level of agreement regarding the different frequency of application of the practice between men and women probably for reasons of gender.

**Table 1:** The low-value practices that reached the greatest level of general agreement (total score > 20.0)

Low-value practice	Total score
14. To administer long half-life benzodiazepines for chronic insomnia treatment in individuals over 65 years old	27.8
30. In patients with difficulty maintaining sleep, to use hypnotics without a previous etiological diagnosis	25.1
25. To use benzodiazepines for the treatment of agitation or delirium in elderly individuals	23.2
39. To recommend analgesics (NSAIDs, paracetamol, and others) for more than 15 days per month in primary headaches that do not respond to treatment	22.5
8. To prescribe medications without considering previous treatment, assessing interactions and the degree of adherence to compliance	22.4
5. To prescribe treatment for overactive bladder without excluding other pathologies that may cause similar symptoms	21.1
6. To prescribe opioids for acute disabling low back pain before evaluating and considering other alternatives	20.9
12. To use antipsychotics for the treatment of Generalized Anxiety Disorder	20.9
9. To make clinical decisions in individuals over 75 years old without assessing their functional status	20.7
37. To use nonsteroidal anti-inflammatory drugs (NSAIDs) in individuals with hypertension, heart failure or any cause of CKD. including diabetes	20.5
33. To prescribe proton pump inhibitors as gastroprotection in patients without risk factors for gastrointestinal complications	20.4
34. To use two or more nonsteroidal anti-inflammatory drugs (NSAIDs) simultaneously	20.2

**Table 2:** The low-value practices that reached the greatest level of agreement regarding the different frequency of application of the practice between men and women probably for reasons of gender (mean score of item C > 3.0)

Low-value practice	Mean score of item C^a^
14. To administer long half-life benzodiazepines for chronic insomnia treatment in individuals over 65 years old	5.91
30. In patients with difficulty maintaining sleep, to use hypnotics without a previous etiological diagnosis	5,03
39. To recommend analgesics (NSAIDs, paracetamol, and others) for more than 15 days per month in primary headaches that do not respond to treatment	4.88
5. To prescribe treatment for overactive bladder without excluding other pathologies that may cause similar symptoms	4.79
1. To place a urinary catheter in all patients requiring urine control, except severely ill patients who require strict urine control and cannot guarantee voluntary spontaneous urination	4.52
12. To use antipsychotics for the treatment of Generalized Anxiety Disorder	4.33
25. To use benzodiazepines for the treatment of agitation or delirium in elderly individuals	3.91
6. To prescribe opioids for acute disabling low back pain before evaluating and considering other alternatives	3.88
34. To use two or more nonsteroidal anti-inflammatory drugs (NSAIDs) simultaneously	3.47
18. To perform imaging tests (X-ray, MRI, CT) in patients with acute low back pain without alarm signs	3.44
8. To prescribe medications without considering previous treatment, assessing interactions and the degree of adherence to compliance	3.42
15. To request serological tumor marker tests as population screening (for individuals not belonging to defined risk groups for each type of tumor)	3.33
24. To use acetylsalicylic acid for primary prevention in individuals without cardiovascular disease	3.31
37. To use nonsteroidal anti-inflammatory drugs (NSAIDs) in individuals with hypertension, heart failure, or any cause of CKD, including diabetes	3.25
40. To use opioids as symptomatic treatment for primary headaches	3.25
19. To recommend bed rest in patients with acute or subacute low back pain	3.22
33. To prescribe proton pump inhibitors as gastroprotection in patients without risk factors for gastrointestinal complications	3.16
9. To make clinical decisions in individuals over 75 years old without assessing their functional status	3.06
35. To request CT or MRI for nonspecific neck or low back pain without alarm signs	3.06

^a^Item C: The frequency of application of this practice is different between men and women probably for reasons of gender

For the second questionnaire, we show Tables 1 and 2 to the selected experts, excluding those that reached the greatest level of general agreement from Table 2. We received responses from five of the six professionals who were invited to respond the second questionnaire (Additional file 3). All of the experts thought that all the LVPs disclosed in Table 1 were susceptible to a gender bias in the primary care setting, increasing the risk of a SAAE in the population of a certain sex. In regard to the proposal of including other LVPs, the experts though that the following LVPs should be included in the list: 40, 19*, 24, 15*, 1 and 35* (*mentioned by more than one expert).

Finally, the following LVPs are susceptible to a gender bias in the primary care setting, increasing the risk of a SAAE in the population of a certain sex, agreed by the selected expert professionals:

- 14. To administer long half-life benzodiazepines for the chronic treatment of insomnia, in people over 65 years of age.
- 30. In patients with difficulty staying asleep, to use hypnotics without having a previous etiological diagnosis.
- 25. To use benzodiazepines to treat agitation or delirium in the elderly.
- 39. To recommend analgesics (NSAIDs, paracetamol and others) more than 15 days a month in a primary headache that does not respond to treatment.
- 8. To prescribe drugs without considering previous treatment, evaluate interactions and the degree of adherence to compliance.
- 5. To prescribe treatment for overactive bladder without excluding other pathologies that may cause similar symptoms.
- 6. To prescribe opioids in acute disabling low back pain before evaluating and considering other alternatives.
- 12. To use antipsychotics for the treatment of Generalized Anxiety Disorder in Primary Care.
- 9. Making clinical decisions in people over 75 years of age without having evaluated their functional status.
- 37. To use nonsteroidal anti-inflammatory drugs (NSAIDs) in individuals with hypertension or heart failure or CKD of any cause, including diabetes.
- 33. To prescribe PPIs as gastroprotection in patients without risk factors for gastrointestinal complications.
- 34. Using two or more nonsteroidal anti-inflammatory drugs (NSAIDs) simultaneously.
- 18. To perform imaging tests (X-ray, MRI, CT) in patients with acute low back pain without warning signs.
- 40. To use opiates as symptomatic treatment of primary headache.
- 19. To recommend bed rest in patients with acute or subacute low back pain.
- 24. To use aspirin for primary prevention in people without cardiovascular disease.
- 15. To request serological tumor markers such as population screening (people who do not belong to the risk groups defined for each type of tumor).
- 1. To remove a bladder catheter in all patients who require urine output control, except seriously ill patients who require strict urine output control and who cannot ensure voluntary spontaneous urination.
- 35. To indicate CT or MRI in nonspecific neck pain or low back pain without alarm signs.

## Discussion

This study identified nineteen LVPs that may lead to SAAEs and can be applied in the primary care setting with varying frequencies between men and women. This study contributes to the literature by providing new insights into the application of LVPs in primary care, specifically addressing gender disparities in relation to unsafe care. To the best of our knowledge, this issue has not been explored in primary care setting before.

While there is no direct evidence base for comparing the results, the findings underscore the need to consider gender differences in healthcare delivery, addressing specific areas of concern, such as the use of benzodiazepines in older adults and the overprescription of analgesics for pain. Previous literature [14, 19–21] have reported that individuals aged 65 to 80, especially women, are being prescribed benzodiazepines inappropriately at an elevated rate. Regarding the prescription of opioids, primary care is the most relevant prescriber [22], and previous studies found that women were more likely to be prescribed analgesics than men and, therefore, gender bias in analgesic treatment might adversely affect women’s health [23]. Other LVPs, such us performing imaging tests in nonspecific low back pain without warning signs, have been identified previously with a higher frequency in women than men [14].

On the other hand, the results showed that, overall, professionals do not agree with the fact that there are LVPs that are applied more frequently in patients of a certain sex, as other studies reported previously. Thus, it seems that healthcare professionals are unaware this fact.

The results of this study should be interpreted with caution due to the limitations inherent in the methodology used. Since identifying the frequency of these practices within a large pool of LVPs, using a quantitative method by reviewing medical records is complex, often requiring manual review by professionals, we opted for a consensus approach to establish an initial identification of LVPs, laying the groundwork for future quantitative studies. A potential limitation of this study was that the LVPs evaluated are based on the ‘Do Not Do’ recommendations established by Spanish scientific societies, and there may be other LVPs recognized in other countries that were not considered in this study. This geographic limitation may affect the generalizability of our findings to international contexts. Regarding the strategy to establish the resulting score for each LVP, the selection of the weights was based on the judgment of the researchers and their previous experience in the field. We acknowledge that the absence of a standard methodology for establishing these weights limits the generalizability and interpretation of our results. Among other study limitations were the snowball sampling method and the moderate response rate of invited professionals, which could introduce selection bias (the target of 50 participants was not achieved). However, it was not observed that the lack of response was in a specific professional profile. Additionally, the selection of experts for the second questionnaire may have subjective influences. Nevertheless, strengths include the inclusion of professionals with extensive experience in both primary care and gender studies, and a focused analysis of specific practices, providing a detailed insight into potential gender biases in primary care.

Further research on the frequency of LVPs in primary care setting is needed. Although it has been assumed that these LVPs may affect patient safety, more evidence is still lacking. When designing decision support tools for safer prescriptions, it would be beneficial to prioritize practices that are both frequently harmful for patients or society (in terms of costs) and those that are theoretically identified as carrying a risk to women’s health and generating inequalities. Studies on therapeutic effort should also consider this gender aspect. It is crucial to consider sex-related differences when planning therapeutic plans. Healthcare professionals may not be aware of the impact of gender biases in their decisions, and this should be included as a significant issue in training and education. Thus, further research is necessary to better understand the frequency and impact of these LVPs. Additionally, it is important to acknowledge the time needed for professionals to implement recommendations. There is a need to prioritize interventions based on factors such as frequency, harmfulness, and societal costs, in order to effectively manage limited resources and ensure efficient implementation of evidence-based practices [24]. Prioritizing evidence-based interventions and awareness of gender differences are important steps toward more equitable and safer patient care.

## Conclusions

The results have significant implications for improving primary care, highlighting specific practices with potential gender biases. The identification of these practices helps to explore a possible gender bias in primary care setting. These findings should inspire future research and actions to effectively investigate gender disparities in healthcare.

### Supplementary Information

Supplementary Material 1.

Supplementary Material 2.

Supplementary Material 3.

## Data Availability

Data are available upon reasonable request.

## Abbreviations

CKD: Chronic Kidney Disease
CT: Computed Tomography
CV: Coefficient of Variation
LVPs: Low-value practices
MRI: Magnetic Resonance Imaging
NICE: National Institute for Health and Care Excellence
NSAIDs: Non-steroidal anti-inflammatory drugs
PPIs: Proton Pump Inhibitors
SAAE: Serious Avoidable Adverse Event
US: United States
