# Supplementary material for: Identification of low-value practices susceptible to gender bias in primary care setting
Source: BMC Prim Care. 2024 Jun 8;25:205. doi: 10.1186/s12875-024-02456-8 (PMC11161995; doi:10.1186/s12875-024-02456-8)
Supplement: Supplementary file 1 — Supplementary Material 1. [file 12875_2024_2456_MOESM1_ESM.docx]

**Additional File 1.** Questionnaire 1.

Please, rate your level of agreement/disagreement from 0 to 10 (where 0 indicated total disagreement; 5, neither agreement nor disagreement; and 10, total agreement) with the following issues for each of the low-value practices indicated below.

A. This LVP is still relatively frequent in primary care setting.

B. This practice could cause a SAE to the patient.

C. The frequency of application of this practice is different between men and women probably for reasons of gender.

1. To place urinary catheter in all patients requiring urine output control, except severely ill patients who require strict urine control and cannot ensure voluntary spontaneous urination.
2. Place a nasogastric tube routinely in all cases of gastrointestinal bleeding.
3. Regularly replace peripheral venous catheters every 72-96 hours.
4. Use vitamin B6, vitamin B12, and folic acid supplements in people with dementia for the prevention or treatment of cognitive impairment when there is no indication or deficiency.
5. Prescribe treatment for overactive bladder without excluding other conditions that may cause similar symptoms.
6. Prescribe opioids for acute disabling lower back pain before evaluating and considering other alternatives.
7. Use intensive therapeutic measures to achieve an HbA1c reduction <7.5% in elderly individuals with multimorbidity, frailty, dependence, and a life expectancy <10 years.
8. Prescribe medications without considering previous treatment, assessing interactions, and the degree of adherence to compliance.
9. Make clinical decisions in individuals over 75 years old without assessing their functional status.
10. Indicate nasogastric or percutaneous gastrostomy tube placement in patients with advanced dementia.
11. Use plasma serotonin level measurement with a diagnostic criterion for depressive disorder.
12. Use antipsychotics for the treatment of Generalized Anxiety Disorder in Primary Care.
13. Prescribe antihistamines for the treatment of panic disorder.
14. Administer long-acting benzodiazepines for the chronic treatment of insomnia in individuals over 65 years old.
15. Request serological tumor markers as population screening (individuals not belonging to defined risk groups for each type of tumor).
16. Treat bronchial asthma with long-acting/life-term bronchodilators without inhaled corticosteroids.
17. In adults with anemia receiving erythropoiesis-stimulating agents, routinely correct to hemoglobin levels above 12 g/dl (adjust dose to desired Hb level between 10 and 12 g/dl).
18. Perform imaging tests (X-ray, MRI, CT) in patients with acute lower back pain without alarm signs.
19. Recommend bed rest in patients with acute or subacute lower back pain.
20. Prescribe fibrates routinely for primary prevention of cardiovascular disease.
21. Use clopidogrel as first-line monotherapy after myocardial infarction.
22. Use thiazolidinediones in diabetic patients with heart failure.
23. Use sulfonylureas in the treatment of elderly patients with renal insufficiency.
24. Use acetylsalicylic acid for primary prevention in individuals without cardiovascular disease.
25. Use benzodiazepines for the treatment of agitation or delirium in elderly individuals.
26. Initiate antihypertensive treatment immediately if an elevated blood pressure level is detected (BP of 140-159/90-99mmHg) and the cardiovascular risk is moderate or low, without cardiovascular, renal, or organic damage.
27. Routinely aim for a blood pressure target below 130/80 in elderly patients with chronic kidney disease (CKD) and proteinuria.
28. Routinely use the combination of a direct renin inhibitor and an angiotensin-converting enzyme inhibitor (ACEI) or angiotensin II receptor antagonist (ARB).
29. Prescribe folic acid or vitamin C supplements specifically for the treatment of anemia in chronic kidney disease (CKD).
30. In patients having difficulty maintaining sleep, use hypnotics without a prior etiological diagnosis.
31. Use drugs with potential extrapyramidal side effects (antiemetics, antivertigo, prokinetics) in patients with Parkinson's disease.
32. Use long-term corticosteroid treatment in patients with multiple sclerosis.
33. Prescribe proton pump inhibitors (PPIs) as gastroprotection in patients without risk factors for gastrointestinal complications.
34. Use two or more non-steroidal anti-inflammatory drugs (NSAIDs) simultaneously.
35. Perform CT or MRI in nonspecific cervical or lumbar pain without alarm signs.
36. Indicate annual electrocardiograms (ECGs) or other cardiac tests for low-risk patients without symptoms.
37. Use non-steroidal anti-inflammatory drugs (NSAIDs) in individuals with hypertension or heart failure or any cause of chronic kidney disease, including diabetes.
38. Prescribe medications other than metformin to achieve an HbA1c <7.5% in the majority of older adults.
39. Recommend analgesics (NSAIDs, paracetamol, and others) for more than 15 days per month in primary headaches that do not respond to treatment.
40. Use opioids as symptomatic treatment for primary headache.
