# Supplementary material for: Identification of low-value practices susceptible to gender bias in primary care setting
Source: BMC Prim Care. 2024 Jun 8;25:205. doi: 10.1186/s12875-024-02456-8 (PMC11161995; doi:10.1186/s12875-024-02456-8)
Supplement: Supplementary file 2 — Supplementary Material 2. [file 12875_2024_2456_MOESM2_ESM.docx]

Supplementary Table 1. Results of the first survey

|  | **A. This LVP is still relatively frequent in primary care setting.** | | | | | | **B. This practice could cause a SAE to the patient** | | | | | | **C. The frequency of application of this practice is different between men and women probably for reasons of gender.** | | | | | | **Total score** |
| --- | --- | --- | --- | --- | --- | --- | --- | --- | --- | --- | --- | --- | --- | --- | --- | --- | --- | --- | --- |
| **LVP** | **Mean** | **% C.V.** | **N** | **< 6** | **6-7** | **> 7** | **Mean** | **% C.V.** | **N** | **< 6** | **6-7** | **> 7** | **Mean** | **% C.V.** | **N** | **< 6** | **6-7** | **> 7** | **(A)+(1.3*B)+(1.6*C)** |
| 1. To place a urinary catheter in all patients requiring urine control, except severely ill patients who require strict urine control and cannot guarantee voluntary spontaneous urination. | 2.55 | 97.84 | 33 | 87.88% | 6.06% | 6.06% | 6.7 | 34.04 | 33 | 21.21% | 30.30% | 48.49% | 4.52 | 74.76 | 33 | 60.61% | 15.15% | 24.24% | 18.49 |
| 1. Routinely to place a nasogastric tube in all cases of digestive bleeding. | 4 | 81.42 | 33 | 63.64% | 15.15% | 21.21% | 6.33 | 35.93 | 33 | 33.33% | 30.30% | 36.37% | 1.94 | 134.24 | 33 | 90.91% | 6.06% | 3.03% | 15.33 |
| 1. Regularly to replace peripheral venous catheters every 72-96 hours. | 2.94 | 110.42 | 33 | 75.76% | 9.09% | 15.15% | 5.61 | 53.06 | 33 | 36.36% | 27.27% | 36.37% | 1.64 | 149.83 | 33 | 90.91% | 6.00% | 3.09% | 12.86 |
| 1. To use vitamin B6. vitamin B12. and folic acid supplements in individuals with dementia for the prevention or treatment of cognitive impairment when there is no indication or deficiency. | 3.82 | 78.20 | 33 | 66.67% | 24.24% | 9.09% | 3.3 | 67.48 | 33 | 84.85% | 12.12% | 3.03% | 2.33 | 105.09 | 33 | 87.88% | 9.09% | 3.03% | 11.84 |
| 1. To prescribe treatment for overactive bladder without excluding other pathologies that may cause similar symptoms. | 5.12 | 39.93 | 33 | 57.58% | 33.33% | 9.09% | 6.42 | 24.53 | 33 | 30.30% | 42.42% | 27.28% | 4.79 | 64.89 | 33 | 54.55% | 24.24% | 21.21% | 21.13 |
| 1. To prescribe opioids for acute disabling low back pain before evaluating and considering other alternatives. | 4.67 | 59.91 | 33 | 57.58% | 21.21% | 21.21% | 7.73 | 21.74 | 33 | 9.09% | 30.30% | 60.61% | 3.88 | 78.64 | 33 | 60.61% | 27.27% | 12.12% | 20.93 |
| 1. To use intensive therapeutic measures to achieve an HbA1c reduction <7.5% in elderly individuals with multimorbidity. frailty. dependence. and a life expectancy <10 years. | 4.21 | 56.41 | 33 | 72.73% | 21.21% | 6.06% | 7.27 | 31.57 | 33 | 15.15% | 27.27% | 57.58% | 2.45 | 103.19 | 33 | 87.88% | 9.09% | 3.03% | 17.58 |
| 1. To prescribe medications without considering previous treatment. assessing interactions. and the degree of adherence to compliance. | 5.48 | 43.32 | 33 | 51.52% | 21.21% | 27.27% | 8.79 | 12.57 | 33 | 0.00% | 12.12% | 87.88% | 3.42 | 84.41 | 33 | 72.73% | 24.24% | 3.03% | 22.38 |
| 1. To make clinical decisions in individuals over 75 years old without assessing their functional status. | 5.42 | 41.08 | 33 | 45.45% | 30.30% | 24.25% | 7.97 | 16.17 | 33 | 6.06% | 27.27% | 66.67% | 3.06 | 94.85 | 33 | 72.73% | 24.24% | 3.03% | 20.68 |
| 1. To indicate nasogastric tube or percutaneous gastrostomy placement in patients with advanced dementia. | 4.3 | 65.76 | 33 | 54.55% | 33.33% | 12.12% | 7.21 | 28.42 | 33 | 21.21% | 30.30% | 48.49% | 2.27 | 111.91 | 33 | 84.85% | 12.12% | 3.03% | 17.31 |
| 1. To use the measurement of plasma serotonin levels as a diagnostic criterion for depressive disorder. | 2 | 119.34 | 33 | 84.85% | 9.09% | 6.06% | 3.76 | 70.13 | 33 | 72.73% | 18.18% | 9.09% | 2.67 | 115.67 | 33 | 75.76% | 15.15% | 9.09% | 11.16 |
| 1. To use antipsychotics for the treatment of Generalized Anxiety Disorder. | 4.15 | 67.20 | 33 | 66.67% | 18.18% | 15.15% | 7.55 | 20.35 | 33 | 9.09% | 36.36% | 54.55% | 4.33 | 76.52 | 33 | 60.61% | 18.18% | 21.21% | 20.89 |
| 1. To prescribe antihistamines for the treatment of panic disorder. | 1.73 | 124.96 | 33 | 90.91% | 9.09% | 0.00% | 6.3 | 35.84 | 33 | 33.33% | 39.39% | 27.28% | 2.88 | 105.61 | 33 | 75.76% | 18.10% | 6.14% | 14.53 |
| 1. To administer long half-life benzodiazepines for chronic insomnia treatment in individuals over 65 years old. | 7.3 | 31.13 | 33 | 15.15% | 21.21% | 63.64% | 8.48 | 13.45 | 33 | 0.00% | 21.21% | 78.79% | 5.91 | 51.05 | 33 | 30.30% | 36.36% | 33.34% | 27.78 |
| 1. To request serological tumor marker tests as population screening (for individuals not belonging to defined risk groups for each type of tumor). | 4.21 | 59.65 | 33 | 66.67% | 18.18% | 15.15% | 4.64 | 60.79 | 33 | 60.61% | 18.18% | 21.21% | 3.33 | 90.86 | 33 | 75.76% | 9.09% | 15.15% | 15.57 |
| 1. To treat bronchial asthma with long-acting bronchodilators without inhaled corticosteroids. | 4.64 | 61.93 | 33 | 63.64% | 12.12% | 24.24% | 5.73 | 36.65 | 33 | 42.42% | 33.33% | 24.25% | 2.61 | 108.62 | 33 | 84.85% | 9.09% | 6.06% | 16.27 |
| 1. In adults with anemia receiving erythropoiesis-stimulating agents. routinely correct to Hemoglobin levels above 12 g/dl (adjust dose for desired Hb level between 10 and 12 g/dl). | 2.88 | 93.89 | 33 | 78.79% | 15.15% | 6.06% | 4.67 | 49.77 | 33 | 72.73% | 15.15% | 12.12% | 2.27 | 107.07 | 33 | 93.94% | 6.06% | 0.00% | 12.58 |
| 1. To perform imaging tests (X-ray. MRI. CT) in patients with acute low back pain without alarm signs. | 5.94 | 42.03 | 33 | 45.45% | 21.21% | 33.34% | 5.97 | 35.10 | 32 | 50.00% | 21.88% | 28.12% | 3.44 | 85.58 | 32 | 78.13% | 3.13% | 18.74% | 19.21 |
| 1. To recommend bed rest in patients with acute or subacute low back pain. | 4.22 | 70.74 | 32 | 59.38% | 25.00% | 15.62% | 5.72 | 43.71 | 32 | 43.75% | 34.38% | 21.87% | 3.22 | 81.28 | 32 | 87.50% | 3.13% | 9.37% | 16.81 |
| 1. Routinely to prescribe fibrates for primary prevention of cardiovascular disease. | 3.84 | 65.29 | 32 | 71.88% | 15.63% | 12.49% | 5.81 | 32.17 | 32 | 43.75% | 40.63% | 15.62% | 2.56 | 104.33 | 32 | 84.38% | 12.50% | 3.12% | 15.49 |
| 1. To use clopidogrel as monotherapy as the first-line treatment after a myocardial infarction. | 3.22 | 80.54 | 32 | 81.25% | 9.38% | 9.37% | 5.63 | 34.64 | 32 | 46.88% | 37.50% | 15.62% | 2.34 | 112.50 | 32 | 84.38% | 12.50% | 3.12% | 14.28 |
| 1. To use thiazolidinediones in diabetic patients with heart failure. | 3.41 | 58.40 | 32 | 81.25% | 15.63% | 3.12% | 7.13 | 26.15 | 32 | 1.75% | 25.00% | 73.25% | 2.47 | 104.64 | 32 | 84.38% | 12.50% | 3.12% | 16.63 |
| 1. To se sulfonylureas in the treatment of elderly patients with renal insufficiency. | 3.72 | 61.99 | 32 | 78.13% | 15.63% | 6.24% | 7.34 | 24.20 | 32 | 15.63% | 31.25% | 53.12% | 2.28 | 112.00 | 32 | 87.50% | 9.38% | 3.12% | 16.91 |
| 1. To use acetylsalicylic acid for primary prevention in individuals without cardiovascular disease. | 4.19 | 65.39 | 32 | 65.63% | 25.00% | 9.37% | 7.22 | 19.11 | 32 | 9.38% | 4.75% | 85.87% | 3.31 | 87.98 | 32 | 75.00% | 12.50% | 12.50% | 18.87 |
| 1. To use benzodiazepines for the treatment of agitation or delirium in elderly individuals. | 6.5 | 38.07 | 32 | 21.88% | 40.63% | 37.49% | 8.03 | 19.14 | 32 | 6.25% | 15.63% | 78.12% | 3.91 | 80.09 | 32 | 65.63% | 15.63% | 18.74% | 23.20 |
| 1. To initiate antihypertensive treatment immediately if elevated blood pressure levels are detected (BP 140-159/90-99 mmHg) and cardiovascular risk is moderate or low. without cardiovascular. renal. or organic damage. | 4.5 | 57.47 | 32 | 59.38% | 25.00% | 15.62% | 6.59 | 23.89 | 32 | 21.88% | 53.13% | 24.99% | 2.78 | 95.95 | 32 | 87.50% | 6.25% | 6.25% | 17.52 |
| 1. Routinely to aim for a blood pressure target below 130/80 in elderly patients with chronic kidney disease (CKD) and proteinuria. | 4.66 | 48.00 | 32 | 68.75% | 15.63% | 15.62% | 6.38 | 32.49 | 32 | 21.88% | 46.88% | 31.24% | 2.47 | 107.54 | 32 | 87.50% | 6.25% | 6.25% | 16.91 |
| 1. Routinely to use the combination of a direct renin inhibitor and an angiotensin-converting enzyme inhibitor (ACEI) or angiotensin II receptor antagonist (ARB). | 3.41 | 80.80 | 32 | 71.88% | 18.75% | 9.37% | 6.88 | 29.22 | 32 | 25.00% | 31.25% | 43.75% | 2.13 | 126.09 | 32 | 84.38% | 9.38% | 6.24% | 15.76 |
| 1. To prescribe folic acid or vitamin C supplements specifically for the treatment of anemia in chronic kidney disease (CKD). | 3.09 | 75.78 | 32 | 84.38% | 12.50% | 3.12% | 4.59 | 46.35 | 32 | 68.75% | 25.00% | 6.25% | 2.53 | 114.85 | 32 | 87.50% | 3.13% | 9.37% | 13.11 |
| 1. In patients with difficulty maintaining sleep, to use hypnotics without a previous etiological diagnosis. | 7.16 | 24.75 | 32 | 15.63% | 34.38% | 49.99% | 7.63 | 19.80 | 32 | 9.38% | 34.38% | 56.24% | 5.03 | 66.44 | 32 | 43.75% | 25.00% | 31.25% | 25.13 |
| 1. To use drugs with potential extrapyramidal side effects (antiemetics. antivertigo. prokinetics) in patients with Parkinson's disease. | 4.66 | 41.57 | 32 | 68.75% | 25.00% | 6.25% | 7.28 | 25.36 | 32 | 21.88% | 18.75% | 59.37% | 2.88 | 93.61 | 32 | 87.50% | 6.25% | 6.25% | 18.73 |
| 1. To use long-term corticosteroid treatment in patients with multiple sclerosis. | 3.72 | 79.26 | 32 | 68.75% | 15.63% | 15.62% | 6.72 | 27.07 | 32 | 37.50% | 18.75% | 43.75% | 2.78 | 110.81 | 32 | 81.25% | 6.25% | 12.50% | 16.90 |
| 1. To prescribe proton pump inhibitors as gastroprotection in patients without risk factors for gastrointestinal complications. | 6.78 | 29.50 | 32 | 21.88% | 46.88% | 31.24% | 6.59 | 28.55 | 32 | 18.75% | 50.00% | 31.25% | 3.16 | 88.36 | 32 | 84.38% | 6.25% | 9.37% | 20.40 |
| 1. To use two or more nonsteroidal anti-inflammatory drugs (NSAIDs) simultaneously. | 4.06 | 65.24 | 32 | 68.75% | 18.75% | 12.50% | 8.16 | 16.82 | 32 | 6.25% | 12.50% | 81.25% | 3.47 | 90.80 | 32 | 68.75% | 21.88% | 9.37% | 20.22 |
| 1. To request CT or MRI for nonspecific neck or low back pain without alarm signs. | 4.5 | 69.83 | 32 | 56.25% | 21.88% | 21.87% | 5.03 | 46.54 | 32 | 50.00% | 34.38% | 15.62% | 3.06 | 96.04 | 32 | 81.25% | 9.38% | 9.37% | 15.94 |
| 1. To request annual electrocardiograms (ECGs) or other cardiac tests for low-risk patients without symptoms. | 4.53 | 56.60 | 32 | 59.38% | 25.00% | 15.62% | 3.31 | 73.50 | 32 | 84.38% | 9.38% | 6.24% | 2.56 | 110.98 | 32 | 81.25% | 9.38% | 9.37% | 12.93 |
| 1. To use nonsteroidal anti-inflammatory drugs (NSAIDs) in individuals with hypertension. heart failure. or any cause of CKD. including diabetes. | 5.41 | 40.87 | 32 | 43.75% | 40.63% | 15.62% | 7.59 | 25.46 | 32 | 12.50% | 21.88% | 65.62% | 3.25 | 93.90 | 32 | 75.00% | 15.63% | 9.37% | 20.48 |
| 1. To use medications other than metformin to achieve an HbA1c <7.5% in most older adults. | 5.41 | 41.64 | 32 | 50.00% | 31.25% | 18.75% | 6 | 33.07 | 32 | 37.50% | 40.63% | 21.87% | 2.69 | 99.33 | 32 | 84.38% | 12.50% | 3.12% | 17.51 |
| 1. To recommend analgesics (NSAIDs. paracetamol. and others) for more than 15 days per month in primary headaches that do not respond to treatment. | 5.56 | 38.52 | 32 | 40.63% | 40.63% | 18.74% | 7.06 | 25.88 | 33 | 24.24% | 21.21% | 54.55% | 4.88 | 64.12 | 33 | 48.48% | 30.30% | 21.22% | 22.55 |
| 1. To use opioids as symptomatic treatment for primary headaches. | 2.58 | 96.68 | 33 | 87.88% | 6.06% | 6.06% | 6.78 | 33.19 | 32 | 21.88% | 34.38% | 43.74% | 3.25 | 76.92 | 32 | 78.13% | 18.75% | 3.12% | 16.59 |
