# Supplementary material for: Identification of low-value practices susceptible to gender bias in primary care setting
Source: BMC Prim Care. 2024 Jun 8;25:205. doi: 10.1186/s12875-024-02456-8 (PMC11161995; doi:10.1186/s12875-024-02456-8)
Supplement: Supplementary file 3 — Supplementary Material 3. [file 12875_2024_2456_MOESM3_ESM.docx]

**Additional file 3.** Responses to questionnaire 2

Question 1. Do you consider all of them suitable for inclusion in the prospective study? If not, which one would you exclude and why?

Question2. Would you include any of them? If yes, which one and why?

Participant 1

Response 1. I consider all the proposals suitable, especially those where gender differences in patient outcomes are known in scientific literature. For example, those related to inappropriate prescription, especially in women, of benzodiazepines (which may also increase after crises like those related to COVID-19), pre-prescription of analgesics for various pains or NSAIDs (studies indicate that inappropriate pre-prescription is very common). It is noteworthy that some of these practices have received a lower average in the item of differentiated use between men and women. It would be interesting to explore why since the literature has described existing differences.

Response 2. Firstly, note that some of these are already in the first table (9,39,33) and should not be in this second one. Secondly, it would be interesting to include those related to analgesia or pain management (19, 40), the use of NSAIDs or aspirin in cardiovascular disease (37, 24), and the request for tumor markers in people who do not belong to risk groups (15, for example, inappropriate requests for PSA). I consider them interesting because there is evidence of differences in the management of these pathologies between men and women.

Participant 2

Response 1. I would include them all.

Response 2. I would not include them.

Participant 3

Response 1. Assuming they are all suitable due to the implications for patient safety, recommendation 39, recommending analgesics (NSAIDs, paracetamol, and others) more than 15 days a month in a primary headache that does not respond to treatment, is excluded or reformulated differently due to the daily practice, where in case of no response, other treatments (with or without indication) from other therapeutic groups (tricyclic antidepressants, antiepileptics, or calcium antagonists) are initiated.

Response 2. Inserting a urinary catheter for all patients requiring urine control, except seriously ill patients requiring strict urine control and unable to ensure voluntary spontaneous micturition: Clear bias in favor of men. Women are catheterized less. It would be interesting to study this for the same reason for consultation.

Making clinical decisions in people over 75 years old without assessing their functional status: Analyzing this point can lead us to reflect on health inequities in fragile patients by sex.

Ordering CT or MRI in nonspecific cervicalgia or low back pain without alarm signs: Important to analyze differences in this recommendation because it will provide information on other variables, if requested more in active or unemployed people, and what those differences are by sex. If it is related more to the higher attendance of women to consultations.

Prescribing PPI as gastroprotection in patients without risk factors for gastrointestinal complications: It can also provide indirect information on other variables (attendance, higher alcohol consumption in men...).

Participant 4

Response 1. I would include all the recommendations selected as priorities in the table.

Response 2. Ordering CT or MRI in nonspecific cervicalgia or low back pain without alarm signs: It is an action that is often taken in case of persistent pain and does not improve the patient's situation. It is more common in women because they persist with pain for a longer time, but without alarm signs.

Prescribing PPI as gastroprotection in patients without risk factors for gastrointestinal complications: Normally, women more frequently request the use of PPI with any medication they take, despite being told that it is not necessary.

Recommending bed rest in patients with acute or subacute low back pain: It is a frequent recommendation and tends to delay the patient's recovery. It is necessary to explain that it is not appropriate and falls under the category of "do not do."

Participant 5

Response 1. In my opinion, all of them are inappropriate, and I would not exclude any.

Response 2. The question 35 and 15 are very common and do not add value (the request for MRI due to patient pressure without alarm signs and without waiting for resolution with treatment generates a great deal of inappropriate use, and the inevitable screening with tumor markers that do not contribute continues to be a very frequent element of misuse and pressure on healthcare professionals ("ask for my PSA"). This erroneous concept is so deeply rooted among the population and even among healthcare professionals that it is very difficult to refute ("I requested the PSA and it came out 4.6", "why did you request it?"). In the case of women, gynecological markers are often requested for benign morphological ovarian cysts. These problems affect both primary and hospital care equally.
